# Negative dietary cation and anion difference supplementation of twin-bearing Merino ewes grazing pasture in late gestation did not affect lamb growth or survival

**DOI:** 10.1093/jas/skae205

**Published:** 2024-07-24

**Authors:** Amy Laurel Munn, William H E J van Wettere, Alyce Marie Swinbourne, Ian John Lean, Alice Caroline Weaver

**Affiliations:** Davies Livestock Research Centre, the University of Adelaide, Roseworthy, South Australia 5371, Australia; Davies Livestock Research Centre, the University of Adelaide, Roseworthy, South Australia 5371, Australia; Turretfield Research Centre, South Australian Research and Development Institute, Rosedale, South Australia 5350, Australia; Scibus, Camden, New South Wales 2570, Australia; Turretfield Research Centre, South Australian Research and Development Institute, Rosedale, South Australia 5350, Australia

**Keywords:** calcium, lamb survival, late gestation, magnesium, maternal supplementation, sheep

## Abstract

Each year in Australia, 53% of lamb mortalities are attributed to dystocia, with subclinical maternal calcium deficiencies likely contributing to dystocia rates. A negative dietary cation and anion difference (**DCAD**) diet has increased circulating calcium in sheep. Therefore, this study aimed to investigate the effects of supplementing twin-bearing, grazing ewes with a negative DCAD partial mixed ration (**PMR**) during late gestation on ewe calcium and magnesium concentrations and subsequent lamb growth and survival. On day 120 of gestation (dG), blood samples were collected from 115 twin-bearing Merino ewes and analyzed for glucose, ketone bodies, pH, ionized calcium, and serum calcium and magnesium. On dG 130, ewes were moved into lambing paddocks and placed in the following 2 treatment groups; ewes receiving a positive DCAD PMR (DCAD = 287 mEq/kg DM; *n *= 58) and ewes receiving a negative DCAD PMR (DCAD = −125 mEq/kg DM; *n *= 57) fed as a PMR. On dG 140, a blood and urine sample were collected. The urine was tested for pH. Pasture samples were taken on dG 133 and 149 and tested for DCAD and mineral content. When a lamb was 6 to 18 h old, survival, vigor score, liveweight (**LW**), rectal temperature, blood glucose, and body morphology were recorded. At 10 d of age, lamb LW and survival were recorded and a milk sample was collected from ewes. At 44 d of age, lamb LW and survival were recorded. The DCAD of the pastures across the 6 paddocks ranged from 598 to 893 mEq/kg DM. There were no differences in lamb survival, weight, or viability at any timepoint (*P *> 0.05). There were no differences in mineral status, metabolic state, or acid–base balance between the positive and negative DCAD-supplemented ewes (*P *> 0.05) during supplementation (dG 140). Supplementing a negative DCAD diet to ewes grazing pasture during late gestation did not improve lamb survival. The blood and urine pH of the negative DCAD-supplemented ewes indicated a mild metabolic acidosis was not reached due to the high DCAD of the pastures. Further research needs to take careful consideration of the DCAD of pasture when designing a negative DCAD supplement in order for it to be effective.

## Teaser

This study found that feeding a negative dietary cation and anion difference partial mixed ration to grazing twin-bearing Merino ewes during late gestation did not improve calcium or magnesium concentrations. No improvements in lamb survival, weight, or viability were seen.

## Introduction

Dystocia is defined as either a prolonged birth that requires assistance or a difficult birth due to a prolonged and unassisted delivery. In Australia each year, it is estimated that 7.7 million lambs and approximately 300,000 ewe mortalities result from dystocia, which costs the sheep industry approximately $780 million AUD (Bruce et al., 2021). Dystocia can be caused by multiple factors, making it difficult to predict which ewes will require assistance at birth. Furthermore, in Australian sheep production systems, lambing typically occurs outdoors in pasture or crop-based paddocks. Providing assistance and supervision during lambing is not a viable preventative strategy because farmers typically avoid disrupting the lambing process and for time and labor constraints (Jacobson et al., 2020b). Alternative strategies to reduce the incidence of dystocia, and therefore improve both lamb and ewe survival rates are a priority for the sheep industry. One potential opportunity is to improve the nutrition of the ewe before lambing, by increasing the availability of macro minerals such as calcium and magnesium. Imbalances of these macro minerals in pasture can lead to poor circulating concentrations in ewes which has been linked to dystocia (Friend et al., 2020). Therefore optimizing mineral availability is likely to reduce dystocia rates and may be a viable strategy for the industry to adopt (Jacobson et al., 2020a).

Hypocalcemia is common amongst dairy cows, and increases the risk of dystocia and metabolic disease (Mee, 2008; Zaborski et al., 2009; Kebede et al., 2017) and it is likely that a similar relationship occurs in sheep (Silva and Noakes, 1984; Schlumbohm and Harmeyer, 2003). Calcium plays an important role in smooth muscle contractions and a deficiency can lead to weak myometrial contractions, which contributes to dystocia (Wray and Arrowsmith, 2012). Subclinical calcium and magnesium deficiencies have been identified in pregnant ewes grazing pasture in southern Australia and may contribute to incidences of dystocia (Hocking Edwards et al., 2018; Friend et al., 2020). Providing additional calcium in the form of an industry-standard supplement (e.g., limestone) may be counterproductive as the supplement would work against the homeostatic mechanisms of calcium when mineral imbalances are present (Sykes, 2007). Therefore, development of strategies that improve circulating calcium and magnesium concentrations in pregnant ewes is warranted.

It is common to improve calcium concentrations in dairy cows by feeding a transition diet (last 3 wk of gestation and the first 3 wk of lactation) with a negative dietary cation anion difference (DCAD). The DCAD of a diet refers to the difference in the cations and anions calculated by (Na + K) − (Cl + S) in milliequivalents (mEq). The addition of anions (commonly through acidogenic protein meals) can acidify the diet and cause a shift in the acid–base balance of the animal to a mild acidosis. This shift increases the tissue responsiveness to calcium regulatory hormones, resulting in an increase in blood calcium concentrations through enhancing intestinal absorption of calcium and elevating the excretion of calcium from bone (Vagg and Payne, 1970; Horst et al., 1997; Roche et al., 2003; Goff, 2008). Additionally, a mild metabolic acidosis may influence the skeleton to upregulate glucose and lipid metabolism as it has been identified that the skeleton plays a crucial role in upregulating energy metabolism (Lee et al., 2007) during phases of high metabolic demand, such as lactation (Lean et al., 2014). In contrast, a high DCAD diet can cause metabolic alkalosis which is a risk factor for hypocalcemia. Pastures are typically high in DCAD, hence supplements that are negative in DCAD can lessen the metabolic alkalosis (Oetzel, 2022).

Feeding a negative DCAD diet can improve the overall calcium status of sheep (Espino et al., 2003; Las et al., 2007; Wilkens et al., 2016; Masters et al., 2018; Freitag et al., 2021). Recently, Robertson et al. (2022), reported no improvement in survival of lambs born to grazing ewes fed a negative DCAD supplement in the form of a loose lick in late pregnancy; however, this may have been due to a failure of ewes on some farms to consume the targeted level of supplement (either do to the unpalatability and/or neophobia), and hence the supplement did not alter the mineral status of ewes (Robertson et al. 2022). Therefore, further investigation is warranted to determine if maternal supplementation of a negative DCAD supplement improves lamb survival under grazing conditions.

The aim of the current project is to investigate the effects of a negative DCAD partial mixed ration (**PMR**) on metabolic and mineral status of ewes, and, lamb growth and survival. It is hypothesized that supplementing ewes with a negative DCAD PMR in late gestation increases circulating calcium and magnesium concentrations and improves lamb survival.

## Materials and Methods

This experiment was approved by The Department of Primary Industries and Regions South Australia Animal Ethics Committee (#07/23) and was performed in accordance with the “Australian code for the care and use of animals for scientific purposes 8th edition” (National Health and Medical Research Council, 2013). All animal work was conducted at the South Australian Research and Development Institute’s Turretfield Research Centre, Rosedale, South Australia, Australia (−34.552°S, 138.832°E).

### Animal selection and housing

On day 120 of gestation (dG), 115 synchronized and naturally mated twin-bearing ewes were selected and allocated to dietary treatment groups based on liveweight (**LW**), body condition score (**BCS**), and age (5 to 6 yr old). Ewes received either a positive DCAD PMR (positive PMR; *n *= 58); or a negative DCAD PMR (negative PMR; *n *= 57) group (Tables 1 and 2). The average age of the ewes was 5.3 ± 0.5 yr for the positive DCAD ewes and 5.4 ± 0.5 yr for the negative DCAD ewes. The trial was conducted in 3 replicates (replicate 1; *n *= 27, replicate 2; *n* = 43; and replicate 3; *n* = 45), each mated a week apart. Each dietary treatment group was randomly allocated to one of six 5-ha paddocks, which served as the experimental unit, with similar feed and topography. Paddocks 1 (*n *= 14; replicate 1), 4 (*n *= 21; replicate 2), and 5 (*n *= 23; replicate 3) contained positive PMR groups and paddocks 2 (*n *= 13; replicate 1), 3 (*n *= 22; replicate 2), and 6 (*n *= 22; replicate 3) contained negative PMR groups. Each paddock contained a mixture of annual ryegrass, medic, subterranean clover and barley grass. All ewes previously received a PMR of barley and peas. At dG 120 ewes were transitioned to the experimental PMR by increasing the amount of experimental PMR and decreasing the amount of barley/peas mix both by 80g/head each day until they were fully transitioned to the PMR by dG 130. Ewes were group-fed using feeding trays once per day in the morning. One feed tray per five ewes was provided to ensure sufficient trough space for each ewe and to minimize dominance. Nutritional requirement and feed allocation were determined based on the Agricultural Food and Research Council recommendations (Alderman and Cottrill, 1996) and were adjusted throughout gestation based on ewe LW and dG. The amount of PMR offered was 0.88, 0.96, and 1.04 kg/head on dG 130, 140, and 150, respectively, and supplementation duration was based on a previous similar study (Friend et al., 2018). Ewes were supplied with ad libitum access to drinking water and oaten hay as needed. Feed residuals were not recorded as all groups consumed the entire allocated PMR, except when the ration was wet from heavy rain whereby the ewes did not consume any of the ration (*n *= 3 d).

**Table 1.** Composition of the positive and negative PMR-fed during the trial (kg/t)

Feed ingredient, kg/t AF^1^	Positive PMR	Negative PMR
Ground barley	258.7	263.4
Whole oats	295.6	301.0
Lupins	332.6	338.6
Sugar sucrose	7.4	9.0
Mineral premix	1.5	1.5
Dolomitic limestone	59.1	45.1
BioChlor^2^	22.2	22.6
Magnesium sulfate	—	7.5
Calcium sulfate	—	11.3
Sodium bicarbonate	22.2	—
Magnesium oxide	0.7	—

^1^As fed.

^2^Acidogenic protein meal (BioChlor, Arm & Hammer Animal Nutrition, Princeton, NJ).

**Table 2.** Nutrient analysis for positive and negative PMR during the experiment

	Positive PMR	Negative PMR
DM, %	92.5	91.3
Ash, % DM	4.6	3.8
Fat, % DM	7.3	5.8
CP, % DM	18.3	18.3
ADF, %DM	11.3	12.5
NDF, % DM	26.3	26.3
ME, MJ/kg DM^1^	14.0	13.3
Calcium, g/kg	24.0	14.0
Potassium, g/kg DM	5.1	4.9
Magnesium, g/kg DM	1.9	2.0
Sodium, g/kg DM	7.7	0.64
Phosphorus, g/kg DM	2.8	2.7
Sulfur, g/kg DM	2.0	3.4
Chloride, % DM	0.17	0.24
DCAD, mEq/kg DM^2^	287	−125

^1^ME was calculated by using the following equation: ME = 0.858 + (0.138 × dry organic matter digestibility %) + (0.272 × Fat %).

^2^DCAD was calculated by using the following equation: DCAD = (Na + K) − (Cl + S).

On approximately dG 140, ewes were paint branded on each side to facilitate individual identification in the paddock. From approximately dG 147, ewes were observed twice daily (morning and afternoon) during daylight hours for signs of parturition. Supplementation of experimental PMRs continued until 2.3 ± 0.2 d postpartum. Ewes and their lambs were monitored daily until lambs were 10.0 ± 0.3 d of age.

#### Prepartum measures

On dG 120 and 140, a 10-mL blood sample was collected from ewes via jugular venepuncture using a 10-mL syringe and 18 G 1.5” needle, then dispensed into a 9-mL clot activator vacutainer tube (BD Vacutainer, BD, Belliver Industrial Estate, Plymouth, United Kingdom) and stored on ice upon collection. The remaining 1 mL of blood was immediately measured for ketones, glucose (Abbots Freestyle Optium Neo, Melbourne, Victoria, Australia), pH, and ionized calcium (LAQUAtwin pH-22 compact pH meter and LAQUAtwin Ca-11C bovine calcium meter, Horiba Advanced Techno Co., Ltd, Kyoto, Japan). A 30-mL urine sample was collected on dG 140 via transient apnea (Benech et al., 2015) into a 70-mL plastic container (Sardsted. AG & Co., KG, Germany) and immediately measured for pH (LAQUAtwin pH-22 compact pH meter, Horiba Advanced Techno Co., Ltd). The blood samples were stored at 4 °C for 24 h to allow the sample to clot before being centrifuged at 3,024 × *g* for 15 min. The serum was extracted and divided into 2 aliquots stored at −80 °C.

#### Postnatal measures

At lamb processing (approximately 6 to 18 h postnatal), the date and time of birth (AM/PM), vigor score (1 to 5 where 1 = very energetic/difficult to catch and 5 = moribund (Flinn et al., 2020)) and meconium stain score (1 = no staining, 2 = mild staining, 3 = moderate staining, and 4 = severe staining (Castro-Nájera et al., 2006)) were recorded. Each lamb was ear-tagged with a unique identification, sexed, weighed, and rectal temperature, crown width, and crown to rump length recorded. A 3-mL blood sample was collected via jugular venepuncture using a 5 mL syringe and 21G 1” needle. Blood samples were dispensed into a 5-mL clot activator vacutainer tube (BD Vacutainer, BD, Belliver Industrial Estate) and glucose was measured in the blood remaining in the syringe (Abbots Freestyle Optium Neo). Blood samples were processed and stored in duplicate as described in the prepartum section. At 10.0 ± 0.3 d of age, all lambs were weighed and each ewe was weighed, BCS was recorded and a 30-mL milk sample was collected. Milk samples were obtained by collecting milk across both teats into a 50-mL conical tube. Lastly, at 44.0 ± 0.5 d of age, lambs were weighed.

Lamb necropsies were performed to determine the cause of death (Table 3) of any lambs found dead or euthanized between birth and 10 d of age using methods described by Holst (2004). Cranial and central nervous system hemorrhage were scored as null, minor, moderate, and severe. The 3 categories of dystocia (a, b, and c) will be referred to as dystocia, stillbirth, and birth injury, respectively. These lambs, as well as any lambs missing at each weighing timepoint, were used to calculate lamb survival rates.

**Table 3.** Description of lamb mortality categories (Holst, 2004)

Death category	Description
Dystocia	Edema, cranial and CNS^1^ hemorrhage, not walked, not breathed, ±^2^cleaned, ± assisted.
Stillbirth	Significant cranial and CNS hemorrhage, fat not metabolized, ± breathed, ± assisted.
Birth injury	Significant cranial and CNS hemorrhage, fat metabolized, can have empty stomach, < 7 d old.
SME^3^	Null to minor cranial and CNS hemorrhage, fat metabolized, empty stomach
Misadventure	Lamb missing

^1^Central nervous system.

^2^May or may not occur.

^3^Starvation, mismothering and exposure complex.

#### Biological sample analysis

Ewe serum was analyzed for calcium (reagent; Thermo Fisher) and magnesium (reagent; Randox) via Konelab20Xti (Thermo Scientific, Finland) by the University of Sydney, Veterinary Pathology Diagnostic Services, Sydney, New South Wales, Australia. Ewe milk samples were analyzed for fat, protein, lactose, individual cell count (**ICC**), and solids nonfat (SNF) by National Herd Development, Kyabram, Victoria, Australia. Lamb serum was analyzed for immunoglobulin G (IgG) content via a previously validated radial-immunodiffusion (RID) assay developed at the University of Adelaide’s Veterinary Diagnostic Laboratory (Roseworthy Campus, Roseworthy, South Australia, Australia) with methods described by Brougham et al. (2020). Lamb shape and size were determined by calculating ponderal index (birth weight (kg)/(crown–rump length (m)^3^) and body mass index (birth weight (kg)/(crown–rump length(m)^2^) respectively with equations provided by Baxter et al. (2008).

### Pasture sample processing and analysis

On dG 133 and 149, food on offer (**FOO**) was recorded and pasture samples were collected from each paddock. Pasture samples were obtained by pooled grab samples taken across the paddock and mimicking the height which the ewes were grazing. Pasture samples were dried at 50 °C then analyzed for calcium, magnesium, phosphorous, sodium, potassium, sulfur, chloride, and DCAD (FeedTest, Agrifood Technology, Victoria, Australia). FOO was determined using a pasture ruler (Meat and Livestock Australia, New South Wales, Australia) that measures pasture height and equates to pasture mass. For each paddock, FOO was measured at each corner of the paddock and 2 from the middle of the paddock, for a total of 6 measurements per paddock.

### Sample size estimations

Serum calcium was the outcome of interest in this study and was used to determine sample size. The software G*Power (version 3.1.9.7) was used to estimate sample size. A standard deviation of 0.22 mmol/L was determined from previous studies with serum calcium being one of the outcomes of interest (Munn et al., 2024). The inputs provided into the software consisted of an effect size = 0.4, α = 0.05, and power = 0.8. Based on the output from the software, the total number of ewes per treatment was estimated to be 60.

### Statistical analysis

Stata (StataCorp LLC, College Station, TX) version 18 was used for statistical analysis with a *P* value of ≤0.05 considered significant and >0.05 and <0.1 considered a trend. For all data, paddock was used as the unit of interest, and ewe or lamb, the unit of measurement. A generalized linear mixed (*mixed* Stata) effects model was used to analyze scale variables, a mixed effects logistic regression was used to analyze dichotomous variables (*melogit* Stata), and the lamb survival data was analyzed using a Weibull parametric survival model with random effects (*metstreg* Stata). For ewe data, the dependent variables consisted of blood parameters, urine pH, milk composition, LW, and BCS. The independent variables included ewe age and dietary treatment group (positive and negative). Ewe within paddock within replicate were random effects. Presupplementation measures (dG 130) were fitted as a co-variable in the model when analyzing ewe data during the supplementation timepoint (dG 140). For lamb data, dependent variables consisted of LW, meconium stain score, body mass index, ponderal index, vigor score, survival, cause of death, blood glucose, serum IgG, and rectal temperature. The independent variables included dietary treatment group (positive and negative), ewe age, and sex of lamb. Lamb within ewe, paddock, and replicate were the random effects and lamb age was fitted as a co-variable when analyzing LW data. A total of 19 ewes from the positive PMR group and 18 ewes from the negative PMR group were removed from the trial either due to the ewe did not give birth to twins (positive PMR *n *= 13 and negative PMR *n *= 15) or metabolic disease/mastitis (positive PMR *n *= 6 and negative PMR *n *= 3). Therefore, the analysis is based on 39 ewes per dietary group.

## Results

### Pasture results

Paddock 6 (negative PMR) had less FOO compared with paddocks 1 (positive PMR), 2 (negative PMR), and 4 (positive PMR) (*P *= 0.046) but not with paddocks 3 (negative PMR) and 5 (positive PMR). The DCAD and the potassium-to-sodium ratio of the all pastures were above the recommended concentration for ruminants (Table 4).

**Table 4.** Combined FOO measurements from dG 133 and 149 and mineral content of pasture on dG 133 and 149. FOO presented as mean ± SEM and mineral content presented as actual values

	Positive PMR						Negative PMR					
	P1		P4		P5		P2		P3		P6	
FOO^2^, t DM/ha	1.8 ± 0.2^a^		1.8 ± 0.2^a^		1.4 ± 0.2^ab^		1.7 ± 0.2^a^		1.3 ± 0.2^ab^		1.1 ± 0.2^b^	
dG	133	149	133	149	133	149	133	149	133	149	133	149
Calcium, g/kg	6.6	6.1	7.7	6.9	9.3	7.2	6.2	5.6	8.2	6.1	7.7	6.7
Potassium, g/kg DM	55.0	51.0	55.0	46.0	51.0	49.0	56.0	50.0	58.0	48.0	48.0	47.0
Magnesium, g/kg DM	2.3	2.1	2.3	1.8	2.7	1.5	2.2	1.9	2.1	1.7	1.8	1.8
Sodium, g/kg DM	1.1	1.2	2.3	1.7	2.7	2.2	1.1	0.9	1.7	1.6	1.8	1.7
Phosphorus, g/kg DM	5.7	4.9	5.6	4.7	5.8	3.9	5.5	4.6	5.5	4.7	5.3	4.3
Sulfur, g/kg DM	3.2	2.8	3.6	3.1	4.2	2.8	3.5	3.0	3.9	3.3	3.8	3.0
Chloride, % DM	1.35	1.27	1.41	1.45	1.47	1.70	1.59	1.54	1.46	1.41	1.8	1.71
DCAD, mEq/kg DM^1^^,^^2^	886	833	883	656	739	685	805	701	895	678	572	598
Total DCAD (pasture + PMR)	1,173	1,120	1,170	943	1,026	972	680	576	770	553	447	473
Calcium:phosphorus^3^	1.2	1.2	1.4	1.5	1.6	1.8	1.1	1.2	1.5	1.3	1.5	1.6
Potassium:sodium^3^	50.0	42.5	23.9	27.1	18.9	22.3	50.9	54.3	34.1	30.0	26.7	27.6

^1^DCAD was calculated by using the following equation: DCAD = (Na + K) − (Cl + S).

^2^dG = day of gestation, DCAD = dietary cation and anion difference, FOO = food on offer, P = paddock, PMR = partial mixed ration.

^3^Required level: calcium:phosphorus = 1.1:2.1, potassium:sodium = 5.6:7.1 (Freer et al., 2007).

^ab^Superscripts indicate significant differences (*P* < 0.05) across rows.

### Ewe measures

There were no differences in LW and BCS between positive and negative PMR ewes (Table 5; *P *> 0.05).

**Table 5.** LW and BCS of twin-bearing ewes at dG 120 and 10 d postpartum supplemented with either a positive or negative DCAD PMR. Data presented as mean ± SEM

	Positive PMR	Negative PMR	*P* value
dG 120			
* n*	39	39	
LW, kg	86.8 ± 2.8	86.9 ± 2.9	0.979
BCS, n	3.68 ± 0.04	3.63 ± 0.05	0.466
10 d postpartum			
* n*	38	38	
LW, kg	82.4 ± 2.3	82.0 ± 2.4	0.887
BCS, n	3.57 ± 0.10	3.61 ± 0.10	0.327

dG = day of gestation, BCS = body condition score, LW = liveweight.

Negative PMR-fed ewes had greater ketones (*P *= 0.041) and tended to have greater magnesium presupplementation (dG 120) compared with positive PMR ewes (Table 6; *P *= 0.071). There were no differences in blood or urine pH at any timepoint between positive and negative PMR ewes (Table 7; *P *> 0.05).

**Table 6.** Blood metabolite and mineral concentrations presupplementation (dG 120) and during supplementation (dG 140) of twin-bearing ewes supplemented with either a positive or negative DCAD PMR. Data presented as mean ± SEM and percentage with *n* in parentheses

	Positive PMR	Negative PMR	*P* value
Presupplementation			
* n*	39	39	
Glucose, mmol/L	3.19 ± 0.17	3.04 ± 0.17	0.353
Ketones, mmol/L	0.66 ± 0.13	0.79 ± 0.13	0.041
Ionized calcium, mmol/L	1.04 ± 0.06	1.07 ± 0.06	0.190
Calcium, mmol/L	2.24 ± 0.06	2.18 ± 0.06	0.147
Magnesium, mmol/L	0.91 ± 0.03	0.95 ± 0.03	0.071
Calcium < 2.0 mmol/L^1^, %	7.7 (3)	10.5 (4)	0.576
Ionized calcium < 1.0 mmol/L^2^, %	23.1 (9)	10.5 (4)	0.101
During supplementation			
* n*	39	39	
Glucose, mmol/L	3.52 ± 0.25	3.32 ± 0.25	0.371
Ketones, mmol/L	0.73 ± 0.10	0.85 ± 0.10	0.368
Ionized calcium, mmol/L	1.08 ± 0.07	1.24 ± 0.07	0.122
Calcium, mmol/L	2.22 ± 0.05	2.23 ± 0.05	0.941
Magnesium, mmol/L	0.96 ± 0.02	1.00 ± 0.02	0.200
Calcium < 2.0 mmol/L^1^, %	23.1 (9)	13.2 (5)	0.436
Ionized calcium < 1.0 mmol/L^2^, %	28.2 (11)	2.6 (1)	0.123

^1^Ewes with serum calcium concentrations below 2.0 mmol/L (subclinical deficiency).

^2^Ewes with blood ionized calcium concentrations below 1.0 mmol/L (subclinical deficiency).

**Table 7.** Acid–base balance measures presupplementation (dG 120) and during supplementation (dG 140) of twin-bearing ewes supplemented with either a positive or negative DCAD PMR. Data presented as mean ± SEM

	Positive PMR	Negative PMR	*P* value
Presupplementation			
* n*	39	39	
Blood pH	7.67 ± 0.08	7.67 ± 0.08	0.968
During supplementation			
* n*	39	39	
Blood pH	7.75 ± 0.04	7.73 ± 0.04	0.313
* n*	38	35	
Urine pH	8.10 ± 0.11	7.95 ± 0.12	0.341

There were no differences in milk fat, protein, lactose, SNF, and ICC between positive and negative PMR ewes (Table 8; *P *> 0.05).

**Table 8.** Milk composition at 10 d postpartum of twin-bearing ewes supplemented with either a positive or negative DCAD PMR. Data presented as mean ± SEM

	Positive PMR	Negative PMR	*P* value
*n*	30	29	
Fat, %	8.0 ± 0.4	8.7 ± 0.4	0.210
Protein, %	5.3 ± 0.3	5.1 ± 0.3	0.660
Lactose, %	4.7 ± 0.2	4.8 ± 0.2	0.836
SNF, %	10.9 ± 0.2	10.8 ± 0.2	0.545
ICC, cells/mL	1,422.7 ± 401.6	1,010.7 ± 408.5	0.475

SNF = solids nonfat, ICC = individual cell count.

### Lamb measures

**Figure 1.**
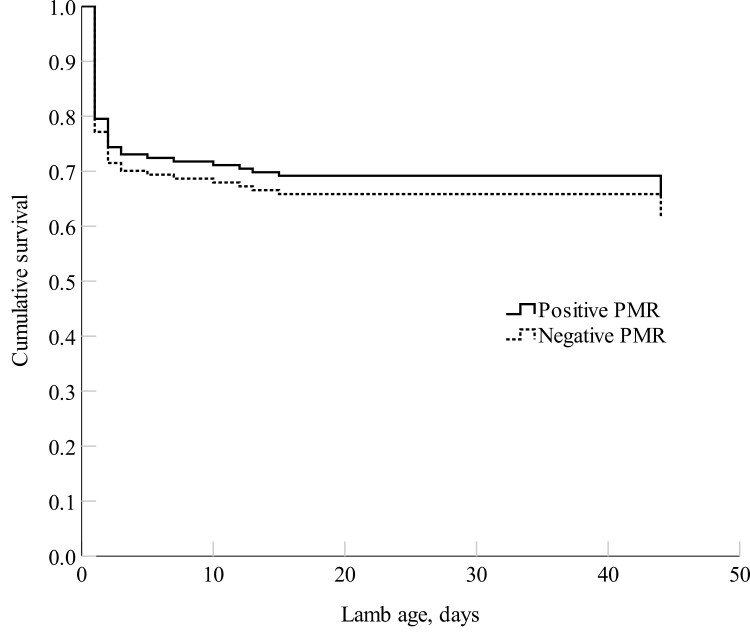
Weibull parametric survival model with random effects of the cumulative lamb survival (proportion) from 6 to 18 h until day 44 of age (average lamb marking age) born to the positive PMR and the negative PMR ewes. The mean survival rate was 65 ± 6% (*n *= 74) for the positive PMR group and 62 ± 6% (*n *= 74) for the negative PMR group.

Lamb survival was similar between the positive and negative PMR groups (Figure 1; *P *= 0.401). There were no differences in the cause of death for lambs born to either positive or negative PMR ewes (Table 9; *P *> 0.05).

**Table 9.** The cause of death of lambs up to 7 d of age that were born to twin-bearing ewes supplemented with either a positive or negative DCAD PMR determined by necropsy results. Data presented as % and *n* in parentheses

	Positive PMR	Negative PMR	*P* value
Deaths up to day 7^1^			
* n*	20	26	
Dystocia, %	20.0 (4)	23.1 (6)	0.802
Stillborn, %	25.0 (5)	34.6 (9)	0.482
Birth injury, %	0.0 (0)	7.7 (2)	0.205
SME, %	55.0 (11)	34.6 (9)	0.167
Total, %	100	100	
All lambs at day 7^2^			
* n*	74	74	
Dystocia, %	5.4 (4)	8.1 (6)	0.512
Stillborn, %	6.8 (5)	12.2 (9)	0.261
Birth injury, %	0.0 (0)	2.7 (2)	0.154
SME, %	14.8 (11)	12.2 (9)	0.631
Total deaths at day 7^3^, %	27.0	35.1	0.287

^1^Percentages consist of deceased lambs only.

^2^Percentages consists of all lambs alive and dead in the trial.

^3^Total deaths at day 7 combined with survival at day 7 = 100% for each treatment group.

Average daily weight gain or LW did not differ at any timepoint for lambs born to either positive or negative PMR ewes (Table 10; *P *> 0.05). Viability measures of lambs born to positive PMR ewes were similar when compared with lambs born to negative PMR ewes (Table 11; *P *> 0.05).

**Table 10:** LW and body size of lambs born to twin-bearing ewes fed either a positive or negative DCAD PMR Data presented as mean ± SEM

	Positive PMR	Negative PMR	*P* value
6 to 18 h			
* n*	69	66	
LW, kg	5.2 ± 0.2	5.2 ± 0.2	0.769
BMI, kg/m^2^	20.5 ± 1.2	18.7 ± 1.2	0.226
PI, kg/m^3^	41.0 ± 3.02	36.9 ± 3.16	0.163
Day 10			
* n*	48	47	
LW, kg	8.1 ± 0.3	7.8 ± 0.3	0.312
Day 44			
* n*	48	46	
LW, kg	16.1 ± 0.5	16.9 ± 0.5	0.269
ADG, g/d	266 ± 10	275 ± 13	0.542

BMI = body mass index, PI = ponderal index, ADG = average daily gain from 6 to 18 h to day 45 of age.

**Table 11:** Lamb viability measures between 6 and 18 h of age that were born to twin-bearing ewes fed either a positive or negative DCAD PMR

	Positive PMR	Negative PMR	*P* value
*n*	67	65	
MSS, 1 to 4	2.2 ± 0.2	2.4 ± 0.2	0.301
Vigor score, 1 to 5	2.7 ± 0.2	2.6 ± 0.2	0.779
Rectal temperature, °C	39.3 ± 0.1	39.3 ± 0.1	0.740
Blood glucose, mmol/L	6.3 ± 1.2	4.4 ± 1.3	0.279
Serum IgG, mg/mL	23.6 ± 2.4	23.0 ± 2.6	0.829

MSS = meconium stain score, IgG = immunoglobulin G.

## Discussion

Our findings in this study did not support the hypothesis that the calcium and magnesium status of twin-bearing ewes grazing pasture supplemented with a negative DCAD PMR will be improved. This was evident as there were no differences in ewe calcium or magnesium concentrations between the positive and negative supplemented groups. There were also no differences in the survival of lambs born to either positive DCAD PMR ewes or negative DCAD PMR ewes. Therefore, there is no support for the hypothesis that twin-bearing ewes grazing pasture supplemented with a negative DCAD PMR will improve lamb survival rates compared to ewes provided a positive DCAD PMR mediated by greater maternal calcium status. Furthermore, maternal negative DCAD supplementation did not improve lamb LW nor lamb viability measures. However, a limitation of our study is that the sample size was estimated to detect differences in mineral status induced by the diet and not lamb survival. Further, the DCAD of the pastures to which the ewes were exposed was extremely high (average of 744 mEq/kg DM; Table 4) which made the total DCAD of the diet positive, despite the negative PMR ewes receiving a negative DCAD supplement. These findings were similar to those reported by Robertson et al. (2022) where providing a negative DCAD supplement in the form of a loose lick (containing magnesium chloride, calcium sulfate, and salt, in the ratio 12.5:32.5:55.0) to ewes grazing pasture did not improve lamb survival or LW; however, in their study, the target concentration of supplementation was not reached. Despite this, there are several contributing factors which may have negated the effectiveness of supplementing a negative DCAD diet to improve lamb survival in grazing conditions.

Negative DCAD diets are commonly used in dairy cattle to prevent clinical and subclinical hypocalcemia by shifting the acid–base balance of the animal to a mild acidosis (Gaynor et al., 1989). This shift improves the calcium status of the animal by increasing tissue responsiveness to parathyroid hormone, an essential hormone in calcium homeostasis, to increase mobilization of calcium from bone (Block, 1984; Gaynor et al., 1989; Phillippo et al., 1994) and enhancing intestinal absorption of calcium (Oetzel et al., 1991; Phillippo et al., 1994). In nondairy sheep, ewes are more susceptible to subclinical hypocalcemia during late gestation due to the demand for calcium for fetal skeletal mineralization and colostrum production (Freer et al., 2007). Calcium plays a significant role in smooth muscle contractions; therefore, ewes that are subclinically deficient in calcium may be at risk of dystocia in the form of uterine inertia from weak uterine contractions (Jacobson et al., 2020a), which in extreme cases may result in lamb death (Friend et al., 2020). In our study, it was expected that the negative DCAD PMR would induce a mild metabolic acidification, improve the calcium status of ewes and in turn, reduce the incidence of dystocia resulting in fewer dystocia-related lamb deaths. There were no improvements in maternal calcium concentration which may explain the lack of differences seen in lamb survival. It is likely that the lambs that died due to dystocia in this study were not due to maternal subclinical hypocalcemia as the ewes that required assistance at delivery, were due to either fetal–pelvic disproportion (*n* = 1) or fetal malpresentation (*n* = 4) and all had normal calcium concentrations. These types of dystocia can occur regardless of ewe health status (Jacobson et al., 2020a), and improving uterine contraction strength/duration may not provide benefits to severely stuck lambs that require manual manipulation. However, more research is required to provide supporting evidence for this. Additionally, all ewes which had lamb deaths due to the other forms of dystocia; stillbirths (death occurs during or shortly after birth) and birth injury (death within 6 d of birth), had serum calcium concentrations within the normal reference range for sheep (2.12 to 2.87 mmol/L (Paynter, 1996)). This indicates the lamb deaths in this study were not due to sub-clinical hypocalcemia. Although as previously stated, our sample size was too low to detect differences in lamb survival rates. Further studies in paddocks or regions which are more prone to pastures or cereal crops with low calcium may be better suited to test a negative DCAD supplement, however, this may be counterproductive if the pastures are extremely positive in DCAD similar to our study.

Negative DCAD diets shift the acid–base balance of the animal to a mild acidosis which is reflected by the urine pH. A urine pH between 6.2 to 6.8 is required to prevent subclinical hypocalcemia when administering anionic salts in dairy cows (Horst et al., 1997; Goff, 2008). In this study, the urine pH of 7.95 was not acidic enough in the negative DCAD-supplemented ewes. Furthermore, regardless of treatment groups, the majority of ewes had a blood pH > 7.61 (*n *= 60/77) which is consistent with metabolic alkalosis. South-eastern Australian pastures have highly variable DCAD values ranging from 0 to 760 mEq/kg DM (Roche et al., 2000; Hocking Edwards et al., 2018; Robertson et al., 2022). Furthermore, these pastures typically have high potassium concentrations which contribute to the high concentrations of DCAD in pasture and metabolic alkalosis rates (Roche et al., 2000; Hocking Edwards et al., 2018) which our pasture analysis also reflected. This indicates that supplementing will be difficult under grazing conditions without regular pasture testing which may not be cost-effective. Although the ewes were not adequately acidified, acidogenic supplementation may still be beneficial under grazing conditions due to the positive linear relationship of DCAD and hypocalcemia incidence, whereby any reduction in DCAD can reduce rates of hypocalcemia (Lean et al., 2006). Therefore, a negative DCAD supplement may be better suited in areas outside south-eastern Australia where the DCAD of pastures is not extremely high and the addition of a negative DCAD supplement can achieve an overall DCAD value of the diet closer to <100 mEq/kg DM.

In addition to lamb survival, there were also no improvements in lamb weight, weight gain, or viability parameters when ewes were provided with a negative DCAD PMR. This was a similar outcome to a recent study that also found no improvements in lamb weight at marking when supplementing grazing ewes (*n *= 600) with a loose lick (containing magnesium chloride, calcium sulfate, and salt, in the ratio 12.5:32.5:55.0) (Robertson et al., 2022). In contrast, other studies have reported improved lamb growth rates and immunity, and tendency for shorter parturition lengths when calcium and magnesium were supplemented during late gestation until 4 wk postpartum in outdoor pens (Ataollahi et al., 2018, 2020, 2021). In our study, concentrations of magnesium and calcium were not improved, which may explain why there were no differences in parameters that could reflect a longer duration of parturition such as meconium stain score, and lamb viability measures (vigor, rectal temperature, serum IgG, and blood glucose). Meconium stain score is an indicator of oxygen deprivation due to a long and/or stressful birth (Castro-Nájera et al., 2006), yet there was no difference between lambs born to negative DCAD-supplemented ewes and those born to positive DCAD-supplemented ewes suggesting parturition length was not affected. Hypoxic damage during the parturition process as a result of dystocia increases the latency of lambs to suckle (Dwyer, 2003) which can impair the thermoregulatory ability of the lamb and lead to cold exposure (Darwish and Ashmawy, 2011). Lambs in our study were born during winter in cold conditions (mean minimum temperature was 5.4 ± 0.7 °C and mean maximum temperature was 15.3 ± 0.6 °C (Bureau of Meterology, 2023)), and no differences were seen in lamb rectal temperature, blood glucose or lamb serum IgG concentration. This supports the conclusion that parturition duration was unaffected by a negative DCAD supplement. Despite the benefits of calcium and magnesium supplementation on lamb growth and viability seen in previous studies (Ataollahi et al., 2018, 2020, 2021), this may also be a reflection on supplementation period. Our study ceased supplementation at approximately 2 d postpartum whereas the other study supplemented until 4 wk postpartum (Ataollahi et al., 2018, 2020, 2021). Further evaluation is needed, especially in grazing areas with calcium deficiencies, to establish the effectiveness of a negative DCAD supplement on lamb growth and viability.

Calcium and magnesium supplementation in ewes housed in outdoor pens has also been shown to improve the metabolic state of ewes by improving energy regulation as evidenced by reduced non-esterified fatty acids (Ataollahi et al., 2018). Additionally, it has recently been identified that the skeleton can influence energy metabolism through osteocalcin, a hormone derived from bone, upregulating glucose and lipid metabolism (Lee et al., 2007) during periods of high metabolic demand (Lean et al., 2014). Negative DCAD rations fed prepartum in dairy cattle stimulate bone turnover and thus potentially can indirectly improve concentrations of osteocalcin, which in turn, may provide benefits to energy metabolism (Rodney et al., 2018) and bypass the implications of supplementing calcium as supplementing calcium alone works against the homeostatic mechanisms of deriving calcium from bone (Sykes 2007). There were no differences in blood glucose or ketone concentrations, or LW and BCS between the negative and positive DCAD-supplemented ewes. This indicates no effect on the ewe’s metabolic state; however, these measures are not reliable indicators of metabolic state. Therefore, further evaluation with reliable measures, such as non-esterified fatty acids, is required. Consequently, based on several meta-analyses in dairy cattle, feeding a negative DCAD diet may reduce feed intake due to the unpalatability of the anionic salt source, however, a palatable diet containing acidogenic protein can improve feed intake and milk yield (Lean et al., 2019; Santos et al., 2019). In our study, intake of the PMR did not appear to differ between the positive and negative DCAD groups, as feed residuals were only present when the feed was wet from rain. This suggests that the negative DCAD PMR was palatable, however, feed intake of pasture and oaten hay was not measured therefore it is unknown what effect the PMR diets had on total feed intake. Milk composition and lamb growth rates were also unaffected which indirectly indicates no effects were seen in milk production, however, to the best of our knowledge, this is the first paper to report the effects of a negative DCAD diet on milk composition in sheep.

## Conclusion

Supplementing a negative DCAD PMR to ewes grazing pasture in late gestation did not improve ewe calcium or magnesium concentrations, or, the survival, weight or viability of their lambs compared with ewes fed a positive DCAD supplement. The blood and urine pH of the negative DCAD-supplemented ewes indicated a mild metabolic acidosis was not reached due to the high DCAD of the pastures. Additionally, the high DCAD of the pastures resulted in the total diet of the negative DCAD-supplemented ewes to be positive, which suggests that negative DCAD supplementation may not be effective with highly positive DCAD pastures unless greater intakes of acidogenic feeds can be achieved. Further research needs to take careful consideration of the DCAD of pasture when designing a negative DCAD supplement in order for it to be effective.

## Abbreviations

ADF: acid detergent fiber
ADG: average daily gain
AF: as fed
BCS: body condition score
BHB: beta-hydroxybutyrate
CP: crude protein
DCAD: dietary cation anion difference
dG: day of gestation
DM: dry matter
G: gauge
IgG: immunoglobulin G
LW: live weight
ME: metabolizable energy
NDF: neutral detergent fiber
PMR: partial mixed ration

## Notes

Conflict of interest statementThe authors declare no conflicts of interest.
